# Electrode-Level Low-Dimensionality Does Not Guarantee Sensor Redundancy: Dual-Dataset, Participant-Grouped Validation of Parsimonious Myoelectric Gesture Decoding

**DOI:** 10.3390/biomimetics11090662

**Published:** 2026-09-15

**Authors:** İsmail Çalıkuşu

**Affiliations:** Biomedical Device Technology Vocational School, Nevsehir Haci Bektas Veli University, Nevsehir 50300, Turkey; ismailcalikusu@nevsehir.edu.tr

**Keywords:** surface electromyography, biomimetic sensing, participant-grouped validation, sensor subset, Myo Armband, sensor re-indexing, electrode-level activation, wearable interface

## Abstract

Electrode-level compressibility may not imply transferable hardware redundancy in biomimetic myoelectric interfaces. This study tested whether sensor-count sufficiency discovered by trial-level analysis survives participant-grouped evaluation. Dataset A comprised 398 archived Myo Armband trials from eight gestures. Dataset B contained 864 one-second trials from 36 participants and six gestures. A timestamp audit identified extensive repeated channel values; Dataset B was therefore analyzed on a conservative 100 Hz grid with 20–45 Hz filtering. Sensor subsets and RBF-SVM parameters were selected exclusively within grouped training data using repeated nested validation. Electrode-level NMF, all 28 fixed six-sensor layouts, cyclic re-indexing, channel-block ablation, participant-cluster bootstrap, PCA, and time-domain-only sensitivity analyses were evaluated. Dataset A yielded 97.74% accuracy with six sensors and 97.93% with eight. In Dataset B, accuracy was 75.96% ± 7.47% with six sensors and 77.93% ± 6.49% with eight; the paired difference was −1.97 percentage points (corrected 95% CI, −5.50 to 1.56). The participant-cluster bootstrap interval was −3.70 to −0.31 points. Active-gesture accuracy was 71.67% and 74.35%, respectively. All fixed six-sensor layouts averaged 74.59%. Three NMF components reconstructed 89.95% ± 1.78% of held-out-participant normalized RMS patterns, with no nonconverged folds. One-position cyclic re-indexing reduced accuracy to 40.28%; channel-block ablation caused losses of 0.62–6.71 points. Low-dimensional electrode-level RMS structure did not establish removable sensors across unseen users. Compact biomimetic interfaces require registration, adaptation, or equivariant processing before physical sensor reduction.

## 1. Introduction

Hand gestures arise from coordinated activation of multiple forearm and hand muscles and enable grasping, manipulation, communication, rehabilitation, and interaction with artificial or virtual environments. Surface electromyography (sEMG) provides a non-invasive measurement of muscle electrical activity and is therefore widely used for decoding motor intent in prosthetic, assistive, and wearable human–machine interfaces. Recent reviews have summarized the development of sEMG-based hand-gesture recognition systems and highlighted the importance of signal processing, feature representation, classifier selection, and robustness across recording conditions and users [1,2].

Wearable circumferential armbands provide a compact means of sampling forearm muscle activity from multiple locations. Recent studies have demonstrated that multi-channel sEMG armbands can support accurate hand-gesture recognition while enabling compact and wireless implementations [3]. Myo Armband-based systems have likewise been investigated for real-time classification using machine-learning approaches [4]. More broadly, wearable-interface research has emphasized the potential of distributed forearm sensing for hand-gesture recognition while identifying hardware configuration and inter-user variability as continuing challenges [5]. Recent work on embedded EMG acquisition further indicates that channel count, acquisition hardware, signal processing, and computational resources must be considered jointly when developing practical wearable systems [6].

Reliable wearable myoelectric decoding nevertheless remains challenging. sEMG characteristics can change with electrode displacement, limb position, recording conditions, user-specific anatomy, and other sources of inter- and intra-user variability. Experimental studies have shown that limb position and electrode shifts can influence pattern-recognition performance, emphasizing the importance of spatial electrode configuration in myoelectric control [7]. These effects are particularly relevant when evaluating reduced electrode arrays because a channel that appears redundant under one recording configuration may provide useful spatial information under another.

A potential biomimetic rationale for sensor reduction is that coordinated motor behavior can exhibit low-dimensional structure. Recent studies of upper-limb muscle activity have demonstrated that muscle activation can be represented using a limited number of coordinated activation patterns, while time-frequency synergy analyses have further explored low-dimensional representations of muscle activity during wrist movements [8,9]. However, the existence of a low-dimensional activation structure does not necessarily imply that individual physical electrodes are redundant. Surface electrodes measure spatially distributed mixtures of underlying muscle activity, and the mapping between activation patterns and recorded channels can vary with electrode location and participant anatomy. Thus, latent compressibility and physical sensor redundancy represent related but distinct hypotheses.

Previous wearable-sEMG studies have also demonstrated that public datasets and standardized acquisition platforms can support systematic evaluation of gesture-recognition algorithms. In the present study, two publicly available datasets are used: the archived Myo Armband dataset [10] and the UCI EMG gesture dataset containing explicit participant information [11]. These datasets differ in acquisition characteristics, gestures, sampling representation, and feature construction and are therefore treated as complementary evaluation resources rather than numerically matched replications.

Recent channel-selection research has directly investigated the relationship between low-dimensional muscle-synergy structure and the number of required sEMG channels. An NMF-based channel-selection method used muscle-synergy information to estimate channel importance and evaluate reduced channel subsets with conventional classifiers [12]. Such findings provide a relevant computational basis for investigating sensor economy, but they do not establish that a selected physical subset will remain optimal for previously unseen participants.

Evaluation design is therefore central to interpreting sensor-reduction performance. Cross-validation estimates can have correlated fold errors, and conventional confidence intervals may underestimate uncertainty; nested approaches provide a more appropriate framework when model-selection variability is also of interest [13]. In a sensor-selection problem, this issue is particularly important because selecting channels and evaluating those same channels on overlapping participants can produce optimistic estimates of generalization.

The initial archived-data analysis employed nested trial-level validation and suggested that six of eight numbered Myo sensors could retain the performance of the complete array. The present study evaluates whether this conclusion survives testing in an independent public dataset with explicit participant identifiers [10,11]. Because the two repositories differ in gestures, sampling representation, window duration, feature construction, and participant structure, the second dataset is used to test the underlying sensor-reduction hypothesis rather than to provide a numerically matched replication.

The interpretation of low-dimensional structure also requires care. NMF has been applied to EMG representations to characterize non-negative activation structure and has been used in recent work on EMG-driven modelling and channel-related analyses [14]. However, mathematical components obtained from NMF should not automatically be interpreted as uniquely identified physiological muscle synergies or neural commands. In the present study, NMF is therefore used as a measure of electrode-level activation compressibility rather than as a direct reconstruction of descending motor commands.

Electrode displacement and user variability provide an additional reason to distinguish computational compression from physical sensor reduction. Recent work has investigated robust myoelectric recognition under electrode-array shifts and has proposed approaches based on motor-unit information and spatial robustness [15]. Other studies have focused on reducing the calibration burden associated with changes in electrode position and user conditions [16]. Cross-user domain-adaptation methods have likewise demonstrated the potential of feature alignment and self-training to improve transfer between users [17,18]. These findings suggest that a reduced array intended for calibration-free operation must be evaluated not only for classification accuracy but also for sensitivity to spatial perturbation and participant variation.

Accordingly, this study aimed to: (1) characterize the sensor-count trade-off in archived trial-level data; (2) evaluate reduced sensor configurations using participant-grouped nested validation; (3) quantify electrode-level activation compressibility in unseen participants; (4) compare training-optimized subsets with all fixed six-sensor layouts; and (5) assess sensitivity to cyclic sensor re-indexing and channel-block ablation. The central hypothesis was that, if low-dimensional motor activation translated into transferable physical sensor redundancy, a reduced array would retain eight-sensor performance under participant-grouped evaluation. The results did not support this proposition: electrode-level activation was compressible, but reduced sensor configurations did not establish transferable performance retention. These findings suggest that compact biomimetic interfaces may require anatomical registration, user adaptation, or rotation-aware processing before physical sensor reduction can be considered reliable for unseen-user operation.

## 2. Materials and Methods

### 2.1. Study Design and Public Data Sources

**Figure 1:**
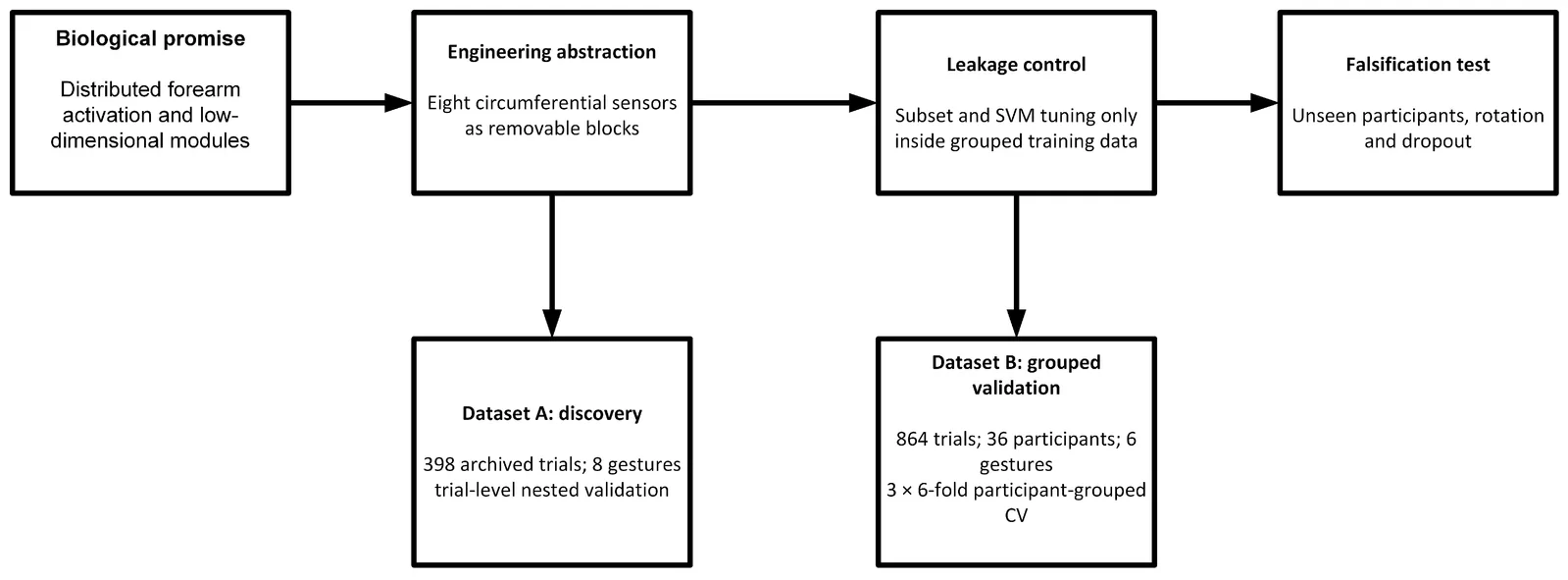
Dual-dataset biomimetic reasoning and validation workflow. Dataset A provides leakage-controlled trial-level discovery; Dataset B tests whether the sensor-count conclusion survives separation of participants. All subset and classifier choices were restricted to grouped training data in Dataset B. *Note: Inference target: sensor-count sufficiency must survive participant grouping; low-dimensional biology does not automatically imply removable electrodes*.

This work was a secondary analysis of two publicly available anonymized sEMG repositories. No new participants were recruited, and no identifiable information was accessed. Dataset A was “EMG Data of the Myo Armband” (Mendeley Data, version 1; DOI: 10.17632/rwbs7645hg.1) [10]. Dataset B was “EMG Data for Gestures” from the UCI Machine Learning Repository (DOI: 10.24432/C5ZP5C) [11], originally described by Lobov et al. [7]. The overall study framework, including the biological premise, engineering abstraction, leakage-control strategy, dataset-specific analyses, and falsification tests, is presented in Figure 1.

Dataset A contained gesture-specific files but no participant, session, arm-side, or anatomical-orientation identifiers. It was therefore retained only as a discovery cohort. Dataset B contained subject-numbered folders for 36 participants, two series per participant, and eight Myo channels. Six gestures performed by every participant were included: rest, fist, wrist flexion, wrist extension, radial deviation, and ulnar deviation. The optional extended-palm class was excluded because it was not performed by all participants [11]. The complementary characteristics, analytical roles, and permitted levels of inference for Datasets A and B are summarized in Table 1.

### 2.2. Dataset A Audit and Discovery Analysis

Every labeled block in Dataset A was required to contain exactly 8 × 100 finite samples. The repository description implied 400 recordings, but only 399 labeled blocks were identifiable because palmar-grasp reading 8 was absent. Hook-grasp trial 21 contained seven 100-sample channels, and one channel was truncated at 78 samples; it was excluded without imputation. The final tensor contained 398 trials. Ninety descriptors were calculated independently per sensor, producing 720 features. The full feature inventory and executed code are included in Supplementary Software S1.

For each sensor count k = 2, …, 8, every complete-sensor subset was evaluated by three-fold inner cross-validation within each outer-training partition. The outer design comprised four repetitions of stratified five-fold validation (20 tests; random state 708). Standardization, subset selection, and RBF-SVM parameter selection were fitted only within the training data. The evaluated (C, γ) pairs were (1, scale), (10, scale), (1, 0.001), (10, 0.001), and (100, 0.001). Dataset A results were interpreted only as discovery because biological grouping variables were unavailable.

### 2.3. Dataset B Parsing, Segmentation, and Signal Processing

All 72 subject-series files were parsed. Contiguous runs with labels 1–6 were identified from the class column. Each file contained two repetitions of the six gestures, yielding four runs per gesture for each participant. A raw-timestamp audit found predominantly 1 ms row intervals but extensive repetition of complete eight-channel values (mean consecutive repetition, 89.15%; file range, 88.32–90.39%). The changing-value intervals were irregular; the channel-wise median was 9 ms, and file-level channel medians ranged from 6 to 12 ms. To avoid treating repeated rows as independent 1 kHz samples or claiming reliable content above 50 Hz, the central 1000 ms interval of each labeled run was interpolated onto a conservative uniform 10 ms grid, producing 100 samples per channel. Central cropping reduced transition contamination and made every observation equal in duration. All 864 candidate runs met the duration criterion; no trial was excluded.

Each trial was filtered independently using a fourth-order zero-phase Butterworth band-pass filter from 20 to 45 Hz. The upper cutoff remained below the 50 Hz Nyquist limit of the conservative analysis grid. This choice limits inference to the retained band and does not assert that the archived row structure preserves the Myo device’s nominal high-frequency content. Filtering was label-independent and performed within each trial. Because only labeled runs were available, filtering could not be applied to a longer continuous recording before central cropping; zero-phase filtering of a 100-sample window can therefore introduce boundary-extension effects. This constraint is considered explicitly in the limitations and should be tested against continuously filtered recordings in prospective work. Ten compact descriptors were then computed per channel: mean absolute value, root mean square, variance, waveform length, difference absolute standard deviation, zero crossings, slope sign changes, skewness, kurtosis, and median frequency [12]. Thresholds for zero crossings and slope sign changes were the larger of $10^{-7}$ and 1% of the trial-level channel standard deviation. The resulting representation contained 80 variables, substantially reducing dimensionality relative to Dataset A. File-level repeat fractions and channel-wise change-interval distributions are provided in Data S1. A prespecified sensitivity analysis repeated the primary six-versus-eight comparison after excluding median frequency, leaving nine time-domain descriptors per channel. The 20–45 Hz band was selected as a conservative consequence of the reconstructed 100 Hz analysis grid: the 45 Hz upper cutoff preserves a guard band below the 50 Hz Nyquist limit, while the 20 Hz lower cutoff attenuates slow baseline and motion-dominated components. This relatively narrow band necessarily excludes higher-frequency sEMG content and may reduce information available to both spectral descriptors and filtered time-domain descriptors; therefore, Dataset B findings should be interpreted as applying to the retained 20–45 Hz content rather than to the full nominal Myo bandwidth. All interpolation, central cropping, and filtering operations were deterministic and applied independently to each trial, without pooling statistics across participants or using outer-test labels or outcomes. The extracted sEMG features and their definitions are summarized in Table 2.

### 2.4. Participant-Grouped Nested Sensor-Subset Optimization

Dataset B used three repetitions of six-fold StratifiedGroupKFold outer validation with random states 708, 1708, and 2708, giving 18 outer tests. All trials belonging to a participant remained together in either training or test data. Each outer test contained six unseen participants. Within every outer-training partition, three-fold stratified grouped validation jointly selected a complete sensor subset and RBF-SVM parameters. Candidate subsets of each size k = 2, …, 8 were exhaustively enumerated. The same prespecified parameter grid used for Dataset A was applied: (C, γ) ∈ {(1, scale), (10, scale), (1, 0.001), (10, 0.001), (100, 0.001)}. Standardization was fitted inside every inner-training fold. Critically, outer-test participants were completely isolated from every data-dependent model-selection step: they were not used to choose sensor subsets, tune C or γ, estimate standardization parameters, rank candidate solutions, or break selection ties. Standardization was re-estimated within each inner-training fold and, after selection, on the complete outer-training participant set only; the untouched outer-test data were transformed once with these training-derived parameters. The adaptive thresholds used for zero crossings and slope-sign changes were trial-local functions of that trial’s own channel standard deviation and did not pool information across participants.

Candidate solutions were ordered deterministically by higher inner balanced accuracy, lower C, lexicographically earlier sensor combination, and predefined γ order. After selection, the winning subset and parameters were refitted on the complete outer-training participants and evaluated once on the untouched outer participants. Accuracy and macro-F1 were calculated for each outer test.

All 28 fixed six-of-eight sensor layouts were evaluated as a label-independent benchmark. For every layout and outer split, SVM parameters were selected only in the same grouped inner folds; layouts were not selected using outer-test outcomes. The distribution across all 28 layouts describes the sensitivity of fixed hardware removal rules. The best observed layout is reported descriptively and is not treated as prospectively selected.

### 2.5. Performance-Retention Inference

The eight-sensor model was the matched reference. Within outer test i, the paired difference was d_i_ = 100 × (accuracy_reduced,i − accuracy_8S,i) percentage points. The exploratory retention margin was Δ = −2 points. Because repeated cross-validation estimates are correlated, uncertainty was estimated using the Nadeau–Bengio corrected resampled variance [13]. For 18 differences and six-fold validation, the test/train ratio was 0.20, and the corrected standard error was SEc = √[(1/18 + 0.20)s^2^d]. A reduced configuration satisfied the exploratory criterion only when the lower two-sided 95% confidence bound exceeded −2 points. The margin was not prospectively registered and does not establish clinical non-inferiority. The six-versus-eight comparison was designated as the primary sensor-reduction contrast; other sensor counts and stress tests were treated as secondary exploratory analyses. As a dependence-robust sensitivity analysis, participant-specific six-versus-eight accuracy differences were averaged across repeated held-out predictions and resampled with a 20,000-replicate participant-cluster bootstrap. The −2 pp margin was chosen as a pragmatic and deliberately stringent engineering tolerance: reducing eight sensors to six removes 25% of the sensing channels, and performance was considered retained only if the data were compatible with an absolute loss smaller than 2 accuracy points. This threshold was not clinically validated or prospectively registered, so it is used only as an exploratory engineering decision rule rather than as a formal clinical non-inferiority margin. The Nadeau–Bengio corrected repeated-cross-validation interval was designated as the primary inferential framework for this retention decision, whereas the participant-cluster bootstrap was used as a dependence-robust sensitivity analysis.

### 2.6. Cross-Participant Electrode-Level Activation Compressibility

To test the biological low-dimensionality premise separately from classification, per-trial RMS values were calculated for the eight filtered channels and normalized by their sum. Nonnegative matrix factorization (NMF) with one to eight components was fitted exclusively on each outer-training participant set [14]. Held-out-participant patterns were transformed using the learned components. Normalized reconstruction fidelity was defined as 1 − ‖P*_test_* − *W_test_H*‖^2^*_F_*/‖*P_test_*‖^2^*_F_*. The denominator is the total squared Frobenius norm of the held-out patterns rather than mean-centered variance; consequently, this quantity must not be interpreted as conventional explained variance. The NMF solver used NNDSVDa initialization (init = “nndsvda”), multiplicative updates (solver = “mu”), Frobenius loss (beta_loss = “frobenius”), a maximum of 5000 iterations, tolerance tol = 1 × 10^−4^, and random_state = 708. The iteration count and convergence status were recorded for every rank and outer fold. This analysis evaluated the compressibility of electrode-level normalized RMS patterns; it did not identify muscles, descending neural commands, or physiological muscle synergies. The 90% level was used only as a descriptive reference, not a validated biological threshold. Training-only principal component analysis (PCA) with held-out-participant reconstruction was added as a linear sensitivity analysis and was evaluated using the same total-energy reconstruction denominator for comparability. The RMS normalization was trial-local: each eight-channel RMS vector was divided only by its own channel sum, so no normalization statistic was estimated from other participants. NMF bases were learned exclusively from outer-training participants; held-out participants were used only for projection onto the learned basis and reconstruction scoring.

### 2.7. Sensor Re-Indexing and Channel-Ablation Stress Tests

Index-registration sensitivity was tested by cyclically shifting all eight test-sensor feature blocks by one to seven positions while leaving the trained indexed model unchanged. This digital re-indexing stress test is not a physical armband-rotation experiment and does not model tissue volume conduction, altered contact, or muscle–electrode geometry. Mean-imputed channel-block ablation replaced one test-sensor feature block with its outer-training mean, which becomes zero after training-derived standardization. It is an idealized missing-feature test rather than a complete model of contact failure, saturation, motion artifact, or additive noise. No test labels were used to adapt the model. A spatial-DFT magnitude representation, mathematically invariant to cyclic sensor re-indexing, was also evaluated with grouped nested parameter selection as a negative control for the trade-off between invariance and spatial specificity. Accordingly, neither perturbation should be interpreted as proving intrinsic physiological importance of a numbered electrode location. Cyclic re-indexing tests dependence on the imposed channel identity/order, whereas mean-imputed block ablation measures dependence of the already-fitted classifier on a feature block under a specific missing-feature surrogate. Correlation among channel features and the distribution shift induced by imputation can affect ablation magnitude; the training-derived mean was used specifically to avoid introducing outer-test information into the replacement value.

### 2.8. Reproducibility and Ethics

Python 3.6 and package versions, random states, parsing rules, trial identifiers, participant-grouped fold assignments, selected subsets, hyperparameters, outer-test predictions, NMF results, and stress-test outputs are provided in Supplementary Software S1 and Data S1. Raw files are not redistributed; permanent source DOIs and licenses are provided. Both datasets were previously released as anonymized public resources. Therefore, no new ethics approval or consent was required for this secondary analysis.

## 3. Results

### 3.1. Data Audit and Observation Structure

Dataset A contained 399 identifiable blocks rather than the described 400. After exclusion of one structurally incomplete block, 398 trials remained. Dataset B contained 72 files, 864 candidate labeled runs, and 864 retained one-second trials. Each of 36 participants contributed four observations for each of six classes. Thus, participant-grouped outer tests contained 144 trials from six unseen participants, and no participant appeared in both training and test data within a fold.

### 3.2. Trial-Level Discovery

In Dataset A, the eight-sensor reference achieved 97.93% ± 1.31% outer-test accuracy. Six sensors achieved 97.74% ± 1.56%, with a paired difference of −0.19 percentage points (corrected 95% CI, −1.26 to 0.89). Under the exploratory −2-point margin, six sensors were the smallest retained count satisfying the discovery criterion. Three sensors still achieved 96.86% ± 1.50%, illustrating how within-repository splitting suggested extensive apparent redundancy. Because participants and sessions were unknown, these findings were treated as a hypothesis rather than hardware evidence.

### 3.3. Participant-Grouped Sensor-Count Validation

The eight-sensor reference achieved 77.93% ± 6.49% accuracy and 77.91% mean macro-F1 across 18 grouped outer tests. Six sensors achieved 75.96% ± 7.47% accuracy and 75.83% macro-F1. Its paired difference from eight sensors was −1.97 points (corrected 95% CI, −5.50 to 1.56), so it did not satisfy the −2-point retention criterion. The participant-cluster bootstrap supported a negative mean difference of −1.97 points (95% CI, −3.70 to −0.31). The bootstrap and corrected repeated-cross-validation intervals quantify different dependence structures: the former supported a negative participant-level mean difference, whereas the primary corrected interval remained compatible with no mean difference and did not establish the retention criterion. Seven sensors achieved 77.78% ± 7.58%, but its interval (difference −0.15 points; corrected 95% CI, −4.42 to 4.11) also crossed the margin. No reduced count met the selected retention rule in participant-grouped testing. These two interval estimates therefore do not conflict with respect to the retention decision: the corrected interval is primary, and its lower bound (−5.50) is below −2 pp, while the bootstrap interval, although entirely below zero, also has a lower bound (−3.70) below −2 pp. Thus, the bootstrap supports a negative participant-level mean difference, but it does not establish the predefined retention requirement either. The participant-grouped outer-test performance and corrected comparisons of each sensor count with the eight-sensor reference are presented in Table 3.

**Figure 2:**
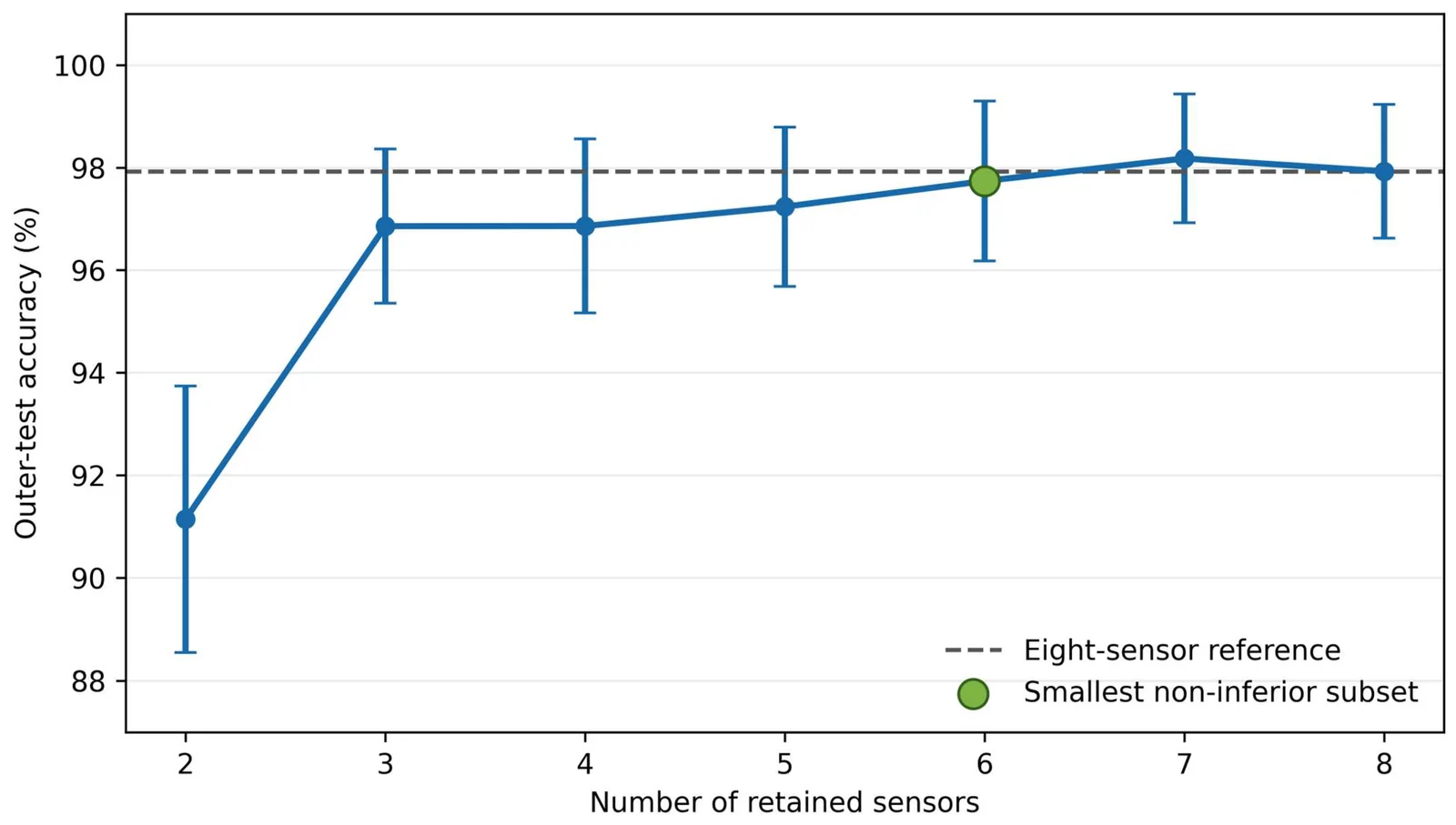
Sensor-count trade-offs under participant-grouped evaluation. Blue markers indicate the mean outer-test accuracy for each number of retained sensors, and error bars represent the standard deviation across correlated outer tests. The dashed horizontal line indicates the mean accuracy of the eight-sensor reference configuration. The green marker identifies the smallest sensor subset that was non-inferior to the eight-sensor reference.

Across all 28 fixed six-sensor layouts, mean accuracy was 74.59%; layout means ranged from 70.14% to 77.20%. The highest descriptive layouts were {S2,S3,S4,S5,S6,S7} and {S2,S4,S5,S6,S7,S8}, each at 77.20%, but neither was prospectively selected, and their corrected intervals crossed the retention margin. Training-only subset selection achieved 75.96%. In the time-domain-only sensitivity analysis, six- and eight-sensor accuracies were 76.23% ± 7.48% and 79.59% ± 7.11%, respectively; the difference was −3.36 points (corrected 95% CI, −6.62 to −0.09). Thus, the principal conclusion did not depend on median frequency. These results show substantial layout dependence and do not establish a universal six-electrode redesign. The sensor-count trade-offs observed in the archived trial-level discovery and participant-grouped evaluation are illustrated in Figure 2.

### 3.4. Electrode-Level Activation Compressibility and Sensor-Selection Stability

**Figure 3:**
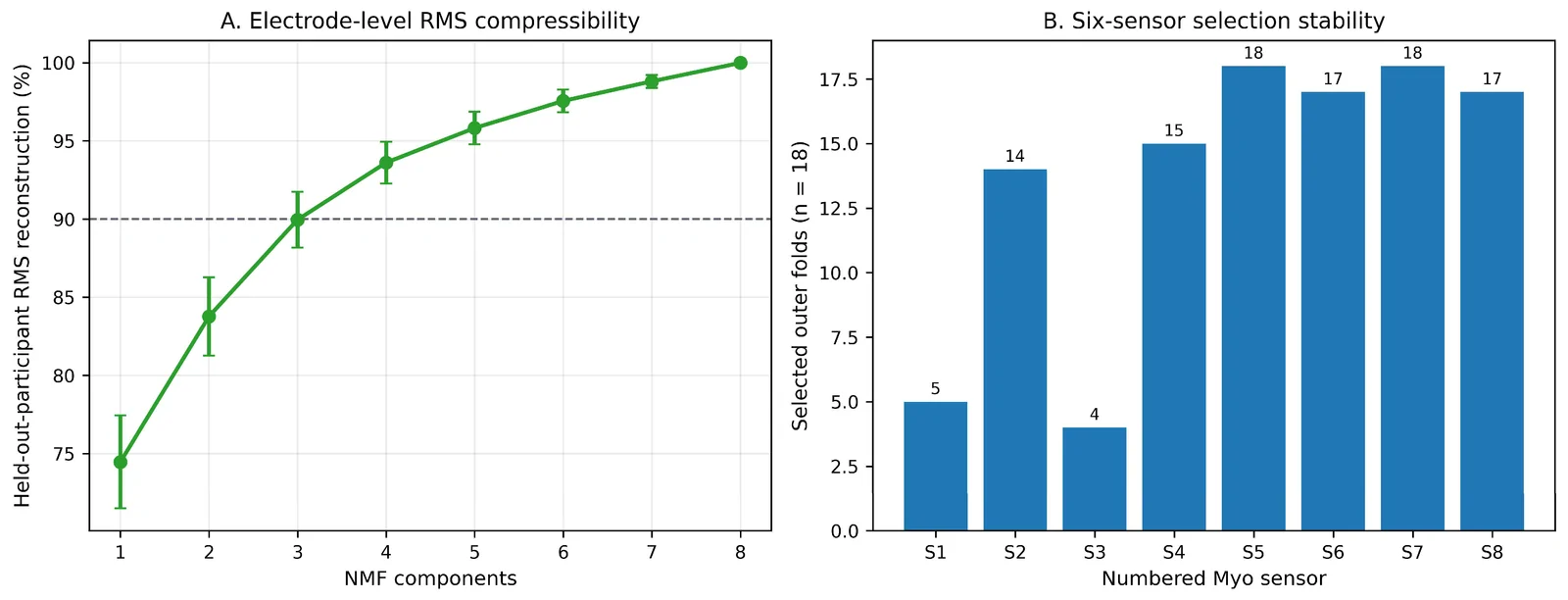
(**A**) NMF reconstruction fidelity for electrode-level normalized RMS patterns in held-out participants. (**B**) selection frequency of numbered channels within grouped six-sensor models. Error bars in panel (**A**) show standard deviations across 18 outer tests.

As shown in Figure 3A, one NMF component reconstructed 74.46% ± 2.97% of the normalized RMS patterns of held-out participants. One NMF component reconstructed 74.46% ± 2.97% of held-out-participant normalized RMS patterns. Two components achieved 83.76% ± 2.51%, and three achieved 89.95% ± 1.78%. Fidelity rose to 93.61% ± 1.33% with four and 95.82% ± 1.05% with five components. All NMF fits converged before 5000 iterations; the maximum observed iteration count was 1540. As a linear sensitivity analysis, held-out-participant PCA fidelity was 83.58%, 90.25%, and 93.76% for one, two, and three components, respectively. These findings demonstrate electrode-level compressibility, not neural-synergy identification.

As shown in Figure 3B, despite this modularity, sensor removal was not interchangeable with latent compression. Despite this modularity, sensor removal was not interchangeable with latent compression. Within six-sensor grouped solutions, S5 and S7 appeared in all 18 outer folds, S6 and S8 in 17, S4 in 15, S2 in 14, S1 in 5, and S3 in 4. The stable inclusion of several locations indicates that latent components remained spatially distributed and required multiple physical samples. Three-sensor solutions always included S5 and S7 and alternated between S2 and S4; they remained 6.21 points below eight sensors (corrected 95% CI, −10.79 to −1.64). NMF fidelity and sensor-selection frequency answer different questions: NMF is unsupervised and quantifies reconstruction of normalized RMS energy patterns, whereas subset selection is supervised and is driven by gesture-discriminative performance in unseen participants. Their divergence therefore indicates that highly reconstructable shared signal structure does not imply that numbered sensor locations are interchangeable for decoding.

### 3.5. Class-Wise Participant-Grouped Performance

**Figure 4:**
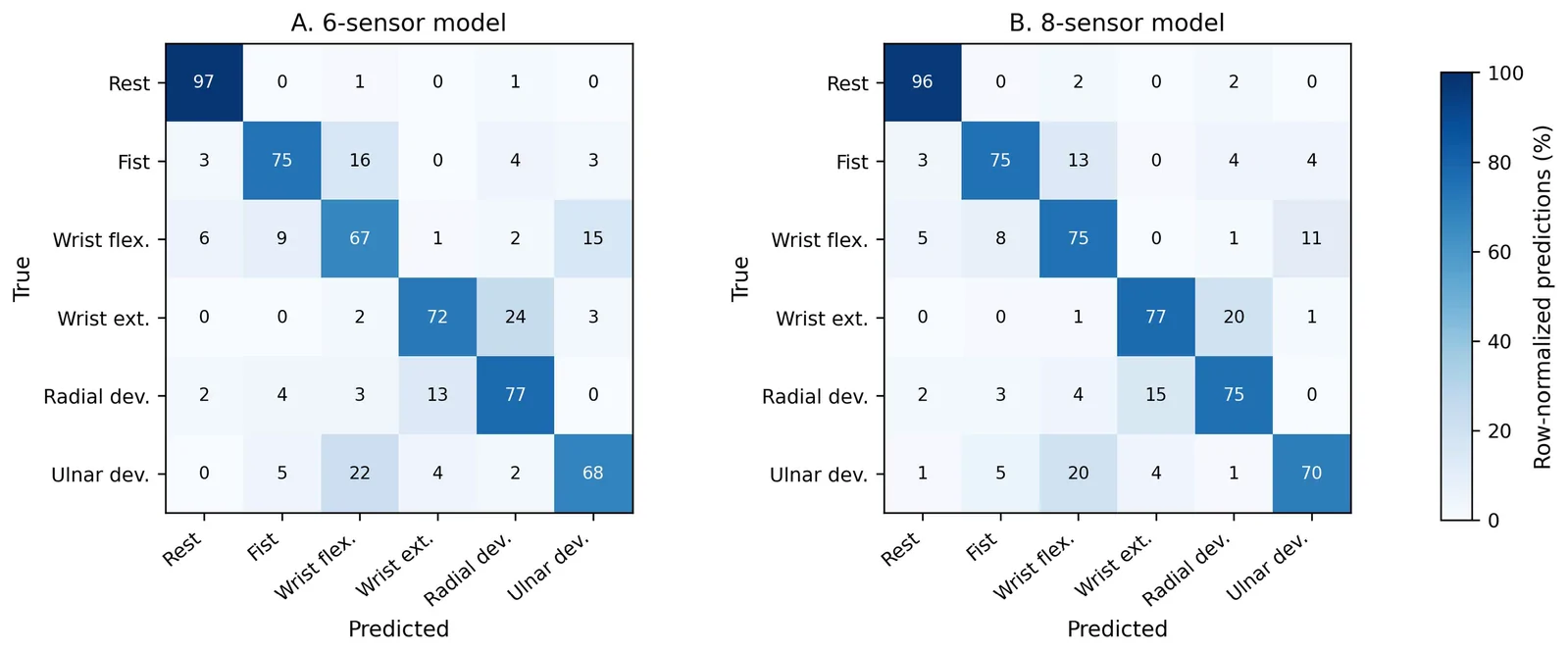
Row-normalized grouped outer-test confusion matrices for (**A**) the six-sensor model and (**B**) the eight-sensor model. Each trial contributed one held-out prediction in each repetition; pooled values are descriptive.

**Figure 5:**
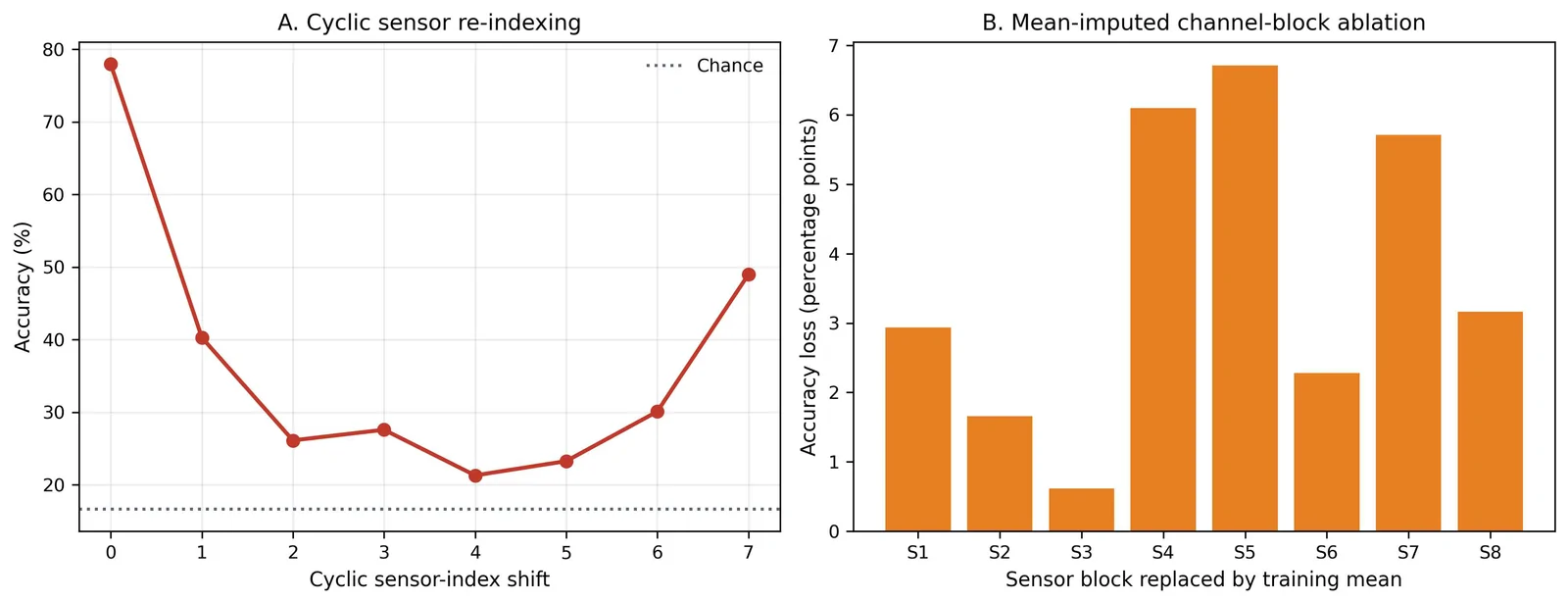
Stress tests for (**A**) cyclic sensor re-indexing and (**B**) mean-imputed channel-block ablation. The dotted horizontal line in panel (**A**) indicates the chance-level accuracy for the six-class classification task (16.67%), whereas the zero-shift point represents the unperturbed eight-sensor reference model. Panel (**A**) digitally shifts complete test-sensor feature blocks and does not represent a physical rotation experiment. Panel (**B**) represents an idealized missing-feature test. These analyses quantify the classifier’s dependence on indexed feature blocks and the selected missing-feature surrogate; they are not direct assessments of physiological electrode importance or actual armband rotation.

Rest was most readily recognized (eight-sensor recall 95.83%). Wrist flexion and ulnar deviation showed residual confusion, with eight-sensor recall of 74.77% and 69.68%, respectively. After excluding rest trials, active-gesture accuracy/balanced accuracy was 74.35% with eight sensors and 71.67% with six. Active-class macro-F1, calculated by averaging F1 only across the five active gesture labels, was 75.02% and 72.41%, respectively. Restricting the label set prevents the absent rest ground-truth class from contributing an artificial zero to the active-class average. These class-specific and active-only results show that overall accuracy was not driven solely by rest and that sensor-count effects varied across movements. Detailed pooled class-wise recall and F1 scores are presented in Table 4, while the corresponding row-normalized confusion matrices for the six- and eight-sensor models are shown in Figure 4. The cyclic sensor re-indexing and mean-imputed channel-block ablation results are illustrated in Figure 5, and all falsification and robustness analyses are summarized in Table 5.

## 4. Discussion

The principal result is a falsification of the simplest interpretation that low-dimensional motor activation necessarily implies redundant physical electrodes. Trial-level discovery suggested that six sensors could reproduce eight-sensor accuracy, whereas participant-grouped testing did not confirm the same retention claim. At the same time, three NMF components reconstructed approximately 90% of held-out-participant channel activation. Taken together, these observations show that electrode-level RMS compressibility and removable electrodes are not equivalent properties. This distinction is consistent with recent work demonstrating that muscle-synergy information can be used for sEMG channel selection without establishing that the same physical subset is universally optimal across participants [12]. The apparent discrepancy is expected because the NMF analysis used only normalized per-channel RMS amplitudes and optimized unsupervised reconstruction, whereas gesture decoding used a broader set of channel-specific amplitude, waveform, distributional, and frequency descriptors and required class separation across unseen users. A low-rank model can therefore capture shared co-activation and dominant amplitude structure while discarding smaller spatial differences that are disproportionately useful for classification. The present results are consistent with the low-dimensional structure reflecting substantial shared amplitude variability, but they do not prove that it is exclusively amplitude-driven or that it contains no task-relevant information.

The biomimetic implication is therefore more specific than a simple “fewer muscles imply fewer sensors” interpretation. Coordinated motor activity can exhibit low-dimensional structure [8,9], but surface electrodes measure spatially distributed mixtures of underlying muscle activity. The mapping between activation patterns and recorded channels can consequently depend on electrode location and participant anatomy. A wearable interface that seeks to exploit biological dimensionality must therefore preserve the measurable spatial expression of the relevant activation patterns rather than assume that latent dimensionality directly determines the minimum number of physical sensors.

The archived trial-level reference and participant-grouped reference differed substantially, but this numerical gap cannot be attributed solely to validation design because the repositories also differ in gestures, sampling representation, windows, features, and participant information. Dataset A therefore motivates a sensor-reduction hypothesis, whereas Dataset B independently evaluates that hypothesis under unseen-participant separation. The grouped result is the more relevant estimate for calibration-free use within Dataset B. More generally, electrode displacement and limb-position variability have been shown to affect myoelectric pattern recognition, supporting the need to consider spatial robustness when interpreting reduced-array performance [7,15,16].

The sensor-count analysis does not support a universal six-electrode redesign. Six-sensor training-only optimization outperformed the mean across fixed layouts, indicating that data-dependent selection can identify useful locations within the training participants. However, its corrected interval crossed the −2-point boundary, and selected identities varied for peripheral sensors. Thus, the observed advantage of optimized subsets should not be interpreted as evidence for a universally optimal six-sensor arrangement. The defensible engineering conclusion is that the full eight-sensor ring remains preferable for unseen-user use under the present pipeline. Reduced arrays may still be viable for user-specific calibration or in applications where a larger performance loss is acceptable. This interpretation is compatible with recent channel-selection research showing that computationally selected subsets can preserve useful classification performance while leaving open the question of transferability across users [12]. From an engineering perspective, a six-channel implementation would remove 25% of the sensing inputs and could reduce electrode count, analog front-end channels, interconnects, sampled-data throughput, and associated processing or power burden, while simplifying wearable integration. These potential benefits were not measured directly in the present datasets, however, and should therefore be treated as expected design advantages rather than demonstrated system-level gains.

The NMF analysis provides a quantitative description of low-dimensional structure without claiming direct recovery of neural commands or uniquely identified physiological muscle synergies. Three nonnegative components achieved 89.95% normalized reconstruction fidelity across held-out participants with no nonconverged folds, while PCA achieved 93.76% under the same total-energy denominator. Because this denominator is not mean-centered variance, these percentages quantify reconstruction relative to total pattern energy and should not be interpreted as conventional explained-variance ratios. They are mathematical summaries of electrode-level RMS patterns rather than identified muscles, descending neural commands, or uniquely determined physiological synergies. Their relevance to the present study is that they demonstrate substantial compressibility in the recorded activation patterns while the classification experiments simultaneously indicate that the spatial sampling provided by individual electrodes remains important. This interpretation is consistent with recent NMF-based EMG channel-selection work [12,14]. Thus, high NMF reconstruction fidelity should be interpreted as redundancy in representing the dominant electrode-level RMS pattern, not as evidence that specific physical sensor sites can be removed without loss of discriminative information. The uneven six-sensor selection frequencies, together with the broad performance range across all 28 fixed layouts, provide supervised evidence that the useful information is spatially non-interchangeable across numbered channels.

Digital re-indexing sensitivity was severe. A one-position cyclic shift approximately halved accuracy, indicating that the indexed classifier relies strongly on sensor order. This experiment should not be interpreted as a quantitative estimate of physical armband rotation because a digital cyclic permutation does not reproduce changes in skin contact, tissue volume conduction, electrode-to-muscle geometry, or limb orientation. Nevertheless, the result demonstrates that the present feature/classifier pipeline is sensitive to the spatial identity of channels. Recent studies of electrode-array shifts similarly indicate that changes in electrode position can degrade myoelectric pattern recognition and motivate spatially robust or adaptive approaches [15,16]. For this reason, the re-indexing result is evidence of index-registration dependence rather than evidence that any particular physical electrode is intrinsically more important.

The spatial-DFT magnitude control removed index-shift sensitivity by construction but also removed spatial phase information and reduced discrimination. This result illustrates the trade-off between invariance and information preservation: complete invariance to channel rotation can eliminate spatial information that is useful for gesture discrimination. Future work should therefore investigate orientation sensing, anatomical registration, circular-equivariant models, or limited user adaptation rather than assuming that spatial information can simply be discarded. Cross-user domain-adaptation studies provide additional evidence that feature alignment and adaptive strategies can improve transfer without necessarily eliminating spatial information [17,18].

Mean-imputed channel-block ablation losses broadly paralleled selection frequency, particularly for S4, S5, and S7. This identifies dependence on specific sensor feature blocks but does not fully reproduce hardware failure. In a practical controller, channel-quality monitoring, uncertainty estimation, and rapid recalibration would therefore be important complements to sensor reduction. A smaller array provides fewer alternative measurements when a contact is poor or a channel fails, making graceful degradation an important engineering criterion alongside mean classification accuracy. The heterogeneous ablation losses likewise indicate conditional model reliance, not causal physiological importance. Confidence in spatial nonuniformity comes from triangulation across independent observations—selection frequency, the 28 fixed-layout benchmark, and block-ablation behavior—while a prospective retraining experiment with physically omitted electrodes would be required to quantify true hardware-specific importance.

The validation strategy also warrants emphasis. Because cross-validation fold estimates are correlated, conventional uncertainty estimates can be overly optimistic; recent statistical work specifically shows that standard cross-validation confidence intervals can have poor coverage and proposes nested approaches for more reliable variance estimation [13]. The present participant-grouped nested design therefore provides a more conservative assessment of sensor-selection performance than a procedure in which the same participants contribute to both selection and evaluation. Nevertheless, the resulting confidence intervals should be interpreted as estimates under the present dataset and experimental design rather than universal guarantees of deployment performance. For the six-versus-eight retention question, the corrected repeated-cross-validation interval is therefore treated as primary because it matches the repeated grouped validation design and was specified for the retention rule; the participant-cluster bootstrap is interpreted as a complementary sensitivity analysis at the participant level. Both analyses fail to demonstrate the −2 pp retention condition, even though only the bootstrap excludes a zero mean difference.

Several limitations remain. Dataset B contained two series but not multiple days; therefore, the grouped design tests unseen participants rather than long-term re-donning. The archived rows contain extensive repeated values and irregular changing-value intervals; the conservative 100 Hz/20–45 Hz preprocessing reduces but cannot eliminate uncertainty about the source acquisition stream. Moreover, each 100-sample trial was filtered independently because longer continuous labeled recordings were unavailable. Zero-phase boundary extension on such short windows may influence waveform and spectral descriptors; the time-domain-only sensitivity analysis addresses dependence on median frequency but does not eliminate this filtering-boundary uncertainty. The central one-second windows were derived from repository labels and do not reproduce asynchronous onset detection. The simulated cyclic shift re-indexes signals but does not model changes in skin contact, tissue volume conduction, or muscle geometry. The six classes are fewer than the eight archived gestures, so Dataset B is an independent evaluation of the design hypothesis rather than a direct replication of identical movements. The −2-point margin remains exploratory and was not derived from clinical utility. Finally, RBF-SVMs and compact handcrafted features were chosen for interpretability and limited sample size; adaptive, domain-adaptive, or rotation-aware models may produce different trade-offs. The restricted 20–45 Hz band may also remove higher-frequency myoelectric information and thereby alter the magnitude of extracted features and classification performance; consequently, the absolute Dataset B accuracies should not be generalized to full-band recordings. The time-domain-only sensitivity analysis removes dependence on median frequency as a feature, but those time-domain features remain calculated from signals already filtered to 20–45 Hz.

Future work should use anatomically registered, multi-day recordings and predeclare the acceptable accuracy–hardware trade-off. Continuous signals should be filtered before extracting central analysis windows, with an explicit comparison against independent-window filtering to quantify boundary effects. A prospective study should compare universal and participant-specific subsets, record armband orientation, measure electrode-contact quality, and evaluate position identification, rotation-aware adaptation, and cross-user domain adaptation [15,16,17,18]. Full pathway latency, energy consumption, wireless payload, donning time, and user-in-the-loop prosthetic performance should also be reported.

Importantly, future sensor-reduction studies should distinguish three separate objectives: reducing the dimensionality of the recorded signal, selecting informative channels for a particular population, and identifying a physically transferable reduced electrode configuration. The present results indicate that success in the first two objectives does not automatically establish the third.

The present dual-dataset framework therefore provides a conservative template for evaluating biomimetic sensor-economy claims. A proposed reduction should survive participant-grouped validation, leakage-controlled selection, latent-structure analysis, fixed-layout comparisons, and spatial perturbation tests. Under the present conditions, the evidence supports substantial compressibility of electrode-level activation patterns but does not support a universal six-electrode replacement for the eight-sensor ring. The more appropriate interpretation is that biological low-dimensionality may provide a useful basis for computational compression and user-specific channel selection, while transferable physical sensor reduction requires additional mechanisms for anatomical registration, orientation robustness, and cross-user adaptation.

## 5. Conclusions

Low-dimensional structure in electrode-level RMS activation patterns did not guarantee physical sensor redundancy. Although six sensors retained eight-sensor performance in archived trial-level discovery, the result did not satisfy the prespecified exploratory retention rule under participant-grouped evaluation: six-sensor accuracy was 75.96% versus 77.93% with eight sensors, and the corrected interval crossed the −2-point boundary. A participant-cluster bootstrap also indicated a negative mean difference. Three NMF components reconstructed 89.95% of held-out-participant normalized RMS patterns, demonstrating electrode-level compressibility rather than direct motor-synergy recovery. For calibration-free myoelectric decoding in this dataset, preserving circumferential coverage and sensor-index registration was more defensible than minimizing electrode count. Compact biomimetic interfaces should therefore incorporate orientation control, adaptation, or equivariant processing before physical sensor reduction.

## Figures and Tables

**Table 1:** Complementary roles of the two public datasets.

Property	Dataset A: Archived Discovery	Dataset B: Grouped Validation
Source	Mendeley Data, rwbs7645hg.1	UCI dataset 481, C5ZP5C
Device	Myo Armband, 8 channels	Myo Armband, 8 channels
Sampling information	Repository reports 50 samples/s	Millisecond-indexed rows with repeated channel values; analyzed on a conservative 100 Hz grid
Participants	Identifiers unavailable	36 subject folders
Retained observations	398 trials; 8 gestures	864 central 1-s trials; 6 gestures
Validation role	Repeated trial-level discovery	Repeated participant-grouped outer testing
Permitted inference	Within-repository separability	Generalization to held-out participants within this dataset

**Table 2:** Compact features used for participant-grouped validation.

Feature	Definition or Purpose
MAV and RMS	Amplitude and signal-energy summaries
Variance	Within-window dispersion
Waveform length	Sum of absolute first differences
DASDV	Root mean square of first differences
Zero crossings	Sign transitions exceeding an adaptive amplitude threshold
Slope sign changes	Local turning points exceeding the same threshold
Skewness and kurtosis	Distribution asymmetry and excess tail weight
Median frequency	Frequency dividing 20–45 Hz periodogram power into equal halves

**Table 3:** Participant-grouped outer-test performance. Corrected intervals compare each count with eight sensors.

Sensors	Accuracy, Mean ± SD	Macro-F1	Difference (pp)	Corrected 95% CI	−2 pp Retained
2	62.04% ± 7.52%	61.38%	−15.90	−21.47 to −10.32	No
3	71.72% ± 5.96%	71.18%	−6.21	−10.79 to −1.64	No
4	76.54% ± 5.92%	76.32%	−1.39	−5.14 to 2.36	No
5	74.50% ± 7.26%	74.28%	−3.43	−7.54 to 0.67	No
6	75.96% ± 7.47%	75.83%	−1.97	−5.50 to 1.56	No
7	77.78% ± 7.58%	77.66%	−0.15	−4.42 to 4.11	No
8	77.93% ± 6.49%	77.91%	Reference	Reference	Reference

**Table 4:** Pooled class-wise recall and F1 across three repeated grouped out-of-fold predictions per trial (%). Repeated predictions are descriptive and are not independent observations.

Gesture	6S Recall	6S F1	8S Recall	8S F1
Rest	97.45	93.56	95.83	92.41
Fist	74.54	77.59	74.77	78.02
Wrist flexion	67.13	63.74	74.77	69.84
Wrist extension	71.76	75.33	77.31	78.59
Radial deviation	77.31	73.73	75.23	73.86
Ulnar deviation	67.59	71.66	69.68	74.78

**Table 5:** Summary of falsification and robustness analyses.

Analysis	Result	Interpretation
Trial-level 6S retention	−0.19 pp; CI −1.26 to 0.89	Discovery criterion satisfied
Participant-grouped 6S	−1.97 pp; CI −5.50 to 1.56	Retention criterion not confirmed
Participant-cluster bootstrap	−1.97 pp; CI −3.70 to −0.31	Negative subject-level sensitivity estimate
All 28 fixed 6S layouts	Mean 74.59%; range 70.14–77.20%	Strong layout dependence
Three-component NMF	89.95% ± 1.78% fidelity; 0/18 nonconverged folds	Electrode-level RMS patterns were compressible
One-position cyclic re-indexing	40.28% accuracy	Strong sensor-order dependence
Mean-imputed block ablation	0.62–6.71 pp loss	Spatially nonuniform feature dependence
Ring-invariant spatial DFT	54.59% ± 5.11%	Invariance sacrificed discriminative phase

## Data Availability

Dataset A is available from Mendeley Data at https://doi.org/10.17632/rwbs7645hg.1. Dataset B is available from the UCI Machine Learning Repository at https://doi.org/10.24432/C5ZP5C. Source licenses and citation requirements apply.

## Supplementary Materials

The following supporting information can be downloaded at: https://www.mdpi.com/article/10.3390/biomimetics11090662/s1, Software S1: executed parsing, segmentation, filtering, feature extraction, grouped nested optimization, NMF, stress-test, and 600 dpi figure-generation scripts. Data S1: audit records, processed trial features, participant-grouped folds, selected subsets, hyperparameters, outer predictions, class-wise results, NMF fidelity, re-indexing results, channel-ablation results, and software environment. Raw recordings are available from their original repositories and are not redistributed.

## Abbreviations

The following abbreviations are used in this manuscript:

6S	Six-sensor configuration
8S	Eight-sensor configuration
CI	Confidence interval
CV	Cross-validation
DASDV	Difference absolute standard deviation value
DFT	Discrete Fourier transform
EMG	Electromyography
MAV	Mean absolute value
NMF	Nonnegative matrix factorization
NNDSVDa	Nonnegative double singular value decomposition with zeros filled by the mean
PCA	Principal component analysis
pp	Percentage points
RBF	Radial basis function
RMS	Root mean square
SD	Standard deviation
SEc	Corrected standard error
sEMG	Surface electromyography
SVM	Support vector machine
UCI	University of California, Irvine
