# Supplementary material for: Electrode-Level Low-Dimensionality Does Not Guarantee Sensor Redundancy: Dual-Dataset, Participant-Grouped Validation of Parsimonious Myoelectric Gesture Decoding
Source: Biomimetics (Basel). 2026 Sep 15;11(9):662. doi: 10.3390/biomimetics11090662 (PMC13604775; doi:10.3390/biomimetics11090662)
Supplement: Supplementary file 1 [file biomimetics-11-00662-s001.zip › NSSO_EMG_Dual_Dataset_Software_S1_Data_S1/formüller.pdf]

x	Abbreviation	Feature Name	Corrected Mathematical Definition
1	MFL	Maximum Fractal Length	$MFL = \max_k(FL_k)$, where $FL_k = \Sigma \lvert x_{i+1} - x_i \rvert$ for window W_k
2	WA	Willison Amplitude	$WA = \Sigma f(\lvert x_{i+1} - x_i \rvert)$, $i = 1$ to $N-1$<br>where $f(a) = 1$ if $a \geq \theta$, otherwise 0 (19)
3	VARE	Variance of EMG	$VARE = (1/(N-1)) \Sigma (x_i - \mu)^2$, $i = 1$ to N
4	SSI	Simple Square Integral	$SSI = \Sigma x_i^2$, $i = 1$ to N
5	MPR	Myopulse Percentage Rate	$MPR = (100/N) \Sigma I(\lvert x_i \rvert \geq \theta)$, $i = 1$ to N
6	MMAV2	Modified Mean Absolute Value 2	$MMAV2 = (1/N) \Sigma w_i \lvert x_i \rvert$, $i = 1$ to N<br>where $w_i = 1$ for $0.25N \leq i \leq 0.75N$, otherwise $w_i = 0.5$
7	MMAV	Modified Mean Absolute Value	$MMAV = (1/N) \Sigma w_i \lvert x_i \rvert$, $i = 1$ to N<br>where $w_i = 2i/N$ for $1 \leq i \leq N/2$, and $w_i = 2 - 2i/N$ for $N/2 < i \leq N$
8	LD	Log Detector	$LD = \exp[(1/N) \Sigma \ln(\lvert x_i \rvert + \varepsilon)]$, $i = 1$ to N
9	DASDV	Difference Absolute Standard Deviation Value	$DASDV = \sqrt{[(1/(N-1)) \Sigma (x_{i+1} - x_i)^2]}$, $i = 1$ to $N-1$
10	AAC	Average Amplitude Change	$AAC = (1/(N-1)) \Sigma \lvert x_{i+1} - x_i \rvert$, $i = 1$ to $N-1$
11	RMS	Root Mean Square	$RMS = \sqrt{[(1/N) \Sigma x_i^2]}$, $i = 1$ to N
12	WL	Waveform Length	$WL = \Sigma \lvert x_{i+1} - x_i \rvert$, $i = 1$ to $N-1$
13	ZC	Zero Crossing	$ZC = \Sigma f(x_i, x_{i+1})$, $i = 1$ to $N-1$<br>where $f = 1$ if $x_i x_{i+1} < 0$ and $\lvert x_i - x_{i+1} \rvert \geq \theta$, otherwise 0
14	SSC	Slope Sign Change	$SSC = \Sigma f(x_{i-1}, x_i, x_{i+1})$, $i = 2$ to $N-1$<br>where $f = 1$ if $(x_i - x_{i-1})(x_i - x_{i+1}) > \theta$, otherwise 0
15	MAV	Mean Absolute Value	$MAV = (1/N) \Sigma \lvert x_i \rvert$, $i = 1$ to N
16	IEMG	Integrated EMG	$IEMG = \Sigma \lvert x_i \rvert$, $i = 1$ to N
17	AE	Average Energy	$AE = (1/N) \Sigma x_i^2$, $i = 1$ to N
18	VAR	Variance	$VAR = (1/(N-1)) \Sigma (x_i - \mu)^2$, $i = 1$ to N
19	SD	Standard Deviation	$SD = \sqrt{[(1/(N-1)) \Sigma (x_i - \mu)^2]}$, $i = 1$ to N
20	COV	Coefficient of Variation	$COV = \sigma / (\lvert \mu \rvert + \varepsilon)$
21	KURT	Kurtosis	$KURT = [(1/N) \Sigma (x_i - \mu)^4] / \{[(1/N) \Sigma (x_i - \mu)^2]^2 + \varepsilon\}$, $i = 1$ to N
22	SKEW	Skewness	$SKEW = [(1/N) \Sigma (x_i - \mu)^3] / \{[(1/N) \Sigma (x_i - \mu)^2]^{3/2} + \varepsilon\}$, $i = 1$ to N
23	IQR	Interquartile Range	$IQR = Q_3 - Q_1$
24	MAD	Mean Absolute Deviation	$MAD = (1/N) \Sigma \lvert x_i - \mu \rvert$, $i = 1$ to N
25	AR	Autoregressive Coefficients	$x(n) = \Sigma a_j x(n-j) + e(n)$, $j = 1$ to p
26	DAMV	Difference Absolute Mean Value	$DAMV = (1/(N-1)) \Sigma \lvert x_{i+1} - x_i \rvert$, $i = 1$ to $N-1$
27	TM	Temporal Moment	$TM_k = (1/N) \Sigma (x_i - \mu)^k$, $i = 1$ to N
28	VO	V-Order	$VO_p = [(1/N) \Sigma \lvert x_i \rvert^p]^{1/p}$, $i = 1$ to N
29	DVV	Difference Variance Value	$DVV = (1/(N-1)) \Sigma (x_{i+1} - x_i)^2$, $i = 1$ to $N-1$
30	LDAMV	Log Difference Absolute Mean Value	$LDAMV = \ln(DAMV + \varepsilon)$
31	LDASDV	Log Difference Absolute Standard Deviation	$LDASDV = \ln(DASDV + \varepsilon)$
32	CARD	Cardinality	$CARD = \lvert unique(Q(x)) \rvert$<br>where $Q(x)$ denotes quantized EMG samples
33	LCOV	Log Coefficient of Variation	$LCOV = \ln[\sigma/(\lvert \mu \rvert + \varepsilon) + \varepsilon]$
34	LTKEO	Log Teager–Kaiser Energy Operator	$LTKEO = \ln[(1/(N-2)) \Sigma (x_i^2 - x_{i-1}x_{i+1}) + \varepsilon]$, $i = 2$ to $N-1$
35	MSR	Mean Value of Square Root	$MSR = (1/N) \Sigma \sqrt{\lvert x_i \rvert}$, $i = 1$ to N
36	ASS	Absolute Value of Summation of Square Root	$ASS = \lvert \Sigma \sqrt{\lvert x_i \rvert} \rvert$, $i = 1$ to N
37	ASM	Absolute Value of Summation of Exponential Root	$ASM = \lvert \Sigma \exp(\sqrt{\lvert x_i \rvert}) \rvert$, $i = 1$ to N
38	FZC	New Zero Crossing	$FZC = \Sigma I(x_{i-1}x_i < 0$ and $\lvert x_i - x_{i-1} \rvert > \theta)$, $i = 2$ to N
39	EWL	Enhanced Waveform Length	$EWL = \Sigma \lvert x_{i+1} - x_i \rvert^\gamma$, $i = 1$ to $N-1$, $\gamma > 1$
40	EMAV	Enhanced Mean Absolute Value	$EMAV = (1/N) \Sigma w_i \lvert x_i \rvert$, $i = 1$ to N
